# Mice Lacking the Calcitonin Receptor Do Not Display Improved Bone Healing

**DOI:** 10.3390/cells10092304

**Published:** 2021-09-03

**Authors:** Jessika Appelt, Serafeim Tsitsilonis, Ellen Otto, Denise Jahn, Paul Köhli, Anke Baranowsky, Shan Jiang, Melanie Fuchs, Christian H. Bucher, Georg N. Duda, Karl-Heinz Frosch, Johannes Keller

**Affiliations:** 1 Charité–Universitätsmedizin Berlin, Corporate Member of Freie Universität Berlin and Hum-boldt-Universität zu Berlin, Center for Musculoskeletal Surgery (CMSC), Augustenburger Platz 1, 13353 Berlin, Germany; jessika.appelt@charite.de (J.A.); serafeim.tsitsilonis@charite.de (S.T.); ellen.otto@charite.de (E.O.); denise.jahn@charite.de (D.J.); paul.koehli@charite.de (P.K.); melanie.fuchs@charite.de (M.F.); 2 Berlin Institute of Health at Charité–Universitätsmedizin Berlin, Julius Wolff Institute (JWI), Au-gustenburger Platz 1, 13353 Berlin, Germany; christian.bucher@charite.de (C.H.B.); georg.duda@charite.de (G.N.D.); 3 Department of Trauma and Orthopedic Surgery, University Medical Center Hamburg-Eppendorf, 20246 Hamburg, Germany; a.baranowsky@uke.de (A.B.); shan.jiang@stud.uke.uni-hamburg.de (S.J.); k.frosch@uke.de (K.-H.F.); 4 Berlin Institute of Health at Charité–Universitätsmedizin Berlin, BIH Center for Regenerative Therapies (BCRT), Charitéplatz 1, 10117 Berlin, Germany

**Keywords:** calcitonin, calcitonin receptor, bone repair, osteoclasts, osteoblasts

## Abstract

Despite significant advances in surgical techniques, treatment options for impaired bone healing are still limited. Inadequate bone regeneration is not only associated with pain, prolonged immobilization and often multiple revision surgeries, but also with high socioeconomic costs, underlining the importance of a detailed understanding of the bone healing process. In this regard, we previously showed that mice lacking the calcitonin receptor (CTR) display increased bone formation mediated through the increased osteoclastic secretion of sphingosine-1-phosphate (S1P), an osteoanabolic molecule promoting osteoblast function. Although strong evidence is now available for the crucial role of osteoclast-to-osteoblast coupling in normal bone hemostasis, the relevance of this paracrine crosstalk during bone regeneration is unknown. Therefore, our study was designed to test whether increased osteoclast-to-osteoblast coupling, as observed in CTR-deficient mice, may positively affect bone repair. In a standardized femoral osteotomy model, global CTR-deficient mice displayed no alteration in radiologic callus parameters. Likewise, static histomorphometry demonstrated moderate impairment of callus microstructure and normal osseous bridging of osteotomy ends. In conclusion, bone regeneration is not accelerated in CTR-deficient mice, and contrary to its osteoanabolic action in normal bone turnover, osteoclast-to-osteoblast coupling specifically involving the CTR-S1P axis, may only be of minor relevance during bone healing.

## 1. Introduction

Impaired fracture healing, including delayed- and non-unions, still represents an ongoing clinical challenge causing high socioeconomic costs and affecting up to 15% of patients with fractures. Typical complications of impaired bone healing include multiple revision surgeries, chronic pain, and prolonged hospitalization and immobilization, which deeply affect the quality of life in affected patients. Current treatment options are limited in part due to insufficient understanding of the mechanisms governing bone regeneration [1,2]. Consequently, there is an urgent need for the detailed investigation of the bone healing process and the identification and clinical translation of factors positively affecting bone regeneration.

Bone healing can be divided into the inflammation, the repair, and the remodeling phase. First, an acute inflammatory response is triggered through the fractured bone and the rupture of periostal and endostal blood vessels. Hereby, cytokines and growth factors are released, recruiting cells essential for the healing process to the site of injury. Subsequently, the formation of cartilaginous tissue occurs which is later re-vascularized, remodeled, and finally calcified. Similar to normal bone turnover, a fine balance between the activities of bone-resorbing osteoclasts and bone-forming osteoblasts is considered of utmost importance during the entire bone healing process [3,4]. In this regard, we were previously able to show that the hormone calcitonin (CT), primarily expressed by C-cells of the thyroid gland and released into the blood stream, functions as a key regulator in the coupling of bone resorption to bone formation. This was surprising, as countless studies employing salmon CT (sCT), exhibiting an up to 50-fold higher pharmacologic potency than mammalian CT, demonstrated an inhibitory effect on osteoclast function. However, mice lacking endogenous CT or the calcitonin receptor (CTR) unexpectedly displayed high bone mass due to increased bone formation, indicating that the pharmacologic actions of sCT clearly differ from the physiologic function of CT/CTR signaling [5,6,7]. The increased bone formation in global CTR-deficient mice was also observed in mice lacking the CTR specifically in osteoclasts, where CT limits the expression of *Spns2*, which encodes a transmembrane transporter protein facilitating the secretion of sphingosine-1-phosphate (S1P) from osteoclasts. In turn, S1P functions as a paracrine, osteoanabolic molecule, which enhances bone formation by binding to its S1P receptors expressed by osteoblasts [8,9,10,11]. Therefore, CT/CTR signaling functions as a physiologic inhibitor of bone formation and regulates the coupling of bone formation to resorption.

From a clinical point of view and similar to these observations in mice, humans with undetectable CT levels following thyroidectomy display an increased bone mineral density [12]. Most importantly however, CT levels have been reported to be elevated in unconscious polytrauma patients and individuals with fractures, with concentrations even further increasing during the process of fracture healing [13,14]. Therefore, CT/CTR signaling not only regulates bone turnover of the intact skeleton but may also specifically govern the fracture healing process. As the relevance of paracrine osteoclast-to-osteoblast coupling during bone regeneration is largely unknown, our study was designed to investigate fracture healing in mice lacking CTR.

## 2. Materials and Methods

### 2.1. Animals

To assess bone healing, 12-week-old female CTR deficient and C57BL/6J wild-type (WT) mice were utilized. The generation and genotyping of the mutant mice were described previously [7]. Mutant mice were backcrossed at least 7 times to gain a pure C57BL/6J genetic background. In accordance with the 3^R^ principles, data on WT animals served as a positive control group in a previous study with respective experiments conducted 6 weeks prior to the present one, following the identical standardized protocol [15]. The animals were kept at a 12 h light/12 h dark cycle and fed a standard diet and water ad libitum. All animal experiments were approved by the local legal representative animal rights protection authorities (G0277/16) and performed adherent to the policies and principles established by the animal Welfare Act (Federal Law Gazett I, p. 1094) and the national institutes of health guide for care and use of laboratory animals.

### 2.2. Surgical Procedure

Bone injury was induced through a femoral fracture of the left limb using a standardized osteotomy model stabilized with an external fixator as described previously [16]. Briefly, a 2 cm long lateral longitudinal skin incision along an imaginary line from the knee to the hip joint was carried out. The femoral bone was exposed by dissection of the fascia lata and by blunt preparation of the Musc. vastus lateralis and the Musc. biceps femoris, sparing the sciatic nerve. A pin hole was drilled with a hand-drill (diameter: 0.45 mm) proximal to the distal metaphysis of the femur, perpendicular to the longitudinal femoral axis and cortical surface. Thereafter, 3 more drillings for pin placement through the connectors of the external fixator (RISystem, Davos, Switzerland) were conducted, resulting in a fixation of the external fixator construct parallel to the femur. Following rigid fixation, a 0.70 mm osteotomy was performed between both middle pins using a Gigli wire saw (RISystem, Davos, Switzerland). Wound closure was done with an Ethilon 5-0 suture. Bone healing and tissue harvesting was evaluated after 7-, 14-, and 21-days post-surgery (*n* = 6 per genotype and time point), representing the acute inflammation, the soft callus, and the remodeling stage of bone regeneration, respectively. For gene expression analysis, callus tissue was also extracted 3 days post-surgery from additional WT mice with osteotomy.

### 2.3. Serum Analysis

ELISA of serum samples was performed using an ELISA kit (LS-F23047, LSBio, Seattle, WA, USA) specific for mouse CT according to the manufacturer’s instructions.

### 2.4. Gene Expression Analysis

The callus tissue was carefully extracted from the middle of the two central pins of the femoral osteotomy after dissection of the bone from the surrounding soft tissue. The callus tissue was snap frozen, homogenized in Trizol applying an Ultra Turrax (Sigma Aldrich, Merck, Darmstadt, Germany) and the total RNA was isolated using RNeasy Mini Kit (Qiagen, Hilden, Germany). The RNA was reverse transcribed into complementary DNA (cDNA) employing the RevertAid First Strand cDNA Synthesis Kit (ThermoFisher, Waltham, MA, USA). Thereafter, the cDNA was utilized for quantitative real-time PCR (qRT-PCR) using TaqMan Assay-on-Demand primer sets supplied by Applied Biosystems. Gene expression was calculated as fold change expression with *Glyeraldhyde-3-phosphate dehydrogenase* (*Gapdh*) as reference gene.

### 2.5. µCT Analysis

To evaluate the formation of newly formed bone in the osteotomy gap, *µ*CT analyses were conducted using isotropic voxel size of 10.05µm, 80 kV and 124µA (Skyscan 1172F) as previously described in detail [15,17]. In brief, the scan axis coincided with the diaphyseal axis of the femora whereby the analyses were performed on a volume of interests (VOI) compromising 100 slides containing the callus and cortices at 8-bit stacks using CTan v 1.18. 8. Therefore, the resulting grayscale images were segmented using fixed global thresholds (<40 background, 40–100 newly formed bone, >100 cortical bone) allowing the rendering of mineralized callus only. These thresholds were selected based on the histogram of gray-level intensities in accordance with literature data and verified by manual evaluation, excluding unmineralized tissue while preserving the morphology of mineralization. Representative images of µCT-scanned osteotomized bones were visualized with the CTVox software (Bruker, Billerica, MA, USA). For newly formed bone structure visualization within the fracture gap, an adaptive thresholding including thin structures and a morphological escalator delineating between fracture callus and cortical bone was used (CTAn Software, Bruker, Billerica, MA, USA). All data are reported according to the guidelines for tissue imaging by the American Society of Bone and Mineral Research [18].

### 2.6. Histomorphometric Analysis

After 7, 14, and 21 days post-surgery, histological analysis was carried out. In detail, fractured bones were excised with surrounding soft tissue, fixed overnight in 4% PFA and incubated in an accenting sugar gradient (10%, 20% and 30% each for 24 h). The dehydrated bones were placed in molds, immerged with SCEM embedding medium (Section Lab Co Ltd., Hiroshima, Japan) and frozen over cooled hexane (Carl Roth GmbH&CoKG, Karlsruhe, Germany). Following hardening, the bones were longitudinally cut in 5 µm transversal plane sections using a cryotome (Leica CM3050S, Leica Microsystems, Wetzlar, Germany). The sections were mounted on microscope slides using cryofilm (Cryofilm type II C, Section Lab Co Ltd., Hiroshima, Japan). Movat Pentachrome staining was performed for histomorphometric analysis. For that purpose, sections were stained in Alcian Blue (8GS, Chroma), Weigert’s hematoxylin (Merck, Darmstadt, Germany), Brilliant Crocein/Acid Fuchsin (Brilliant Crocein R, Chroma, and Acid Fuchsin, Merck, Darmstadt, Germany), 5% Phosphotungstic acid PTA (Chroma), and Saffron du Gâtinais (Chroma). The stained sections were mounted in Vitro-Clud (Langenbrinck, Emmendingen, Germany). For semi-quantitative assessment of callus bridging, mosaic images of the same samples were taken using the Axioscope 40 (Zeiss, Jena, Germany) and Axiovsion Rel.4.8 software. Histomorphometry was performed through the quantification of different tissue areas in the fracture gap defined as region of interest (ROI) using ImageJ software. Specifically, mineralized tissue and cartilage area were measured in mm^2^ as well as the ratio in percent of these tissues to the total tissue areas comprised of total cortical area, total mineralized bone area, total cartilage area as described previously [15]. Callus bridging of the same samples at day 21 post osteotomy was evaluated with the following scoring: a = complete bridging (all four cortices bridged by callus), b = partial bridging (two to three cortices bridged by callus), c = incomplete bridging (callus present, but no bridging visible), and d = nonunion (rounded cortices, minimal presence of callus). Scoring was performed independently by two blinded reviewers and averages are depicted as stacked bar charts.

### 2.7. Histology

For Trap staining, sections were fixed with 4% PFA, titrated to pH5 using a buffer containing Natriumacetat (Merck, Darmstadt, Germany) and Natriumtartrat-dehydrat (Merck, Darmstadt, Germany), and washed with distilled water. Thereafter, callus sections were stained in the presence of Naphtol AS-MX- Phosphate, Fast Red Violet LB Salt, and N,N-Dimethylformamid. A counterstain was carried out with Mayer’s Hemalaun solution (Merck, Darmstadt, Germany). Osteoclasts were identified as TRAP-positive cells with ≥ 3 nuclei and adherent to the bone surface. Osteoblasts were identified as mononuclear cells adherent to the bone surface with typical cuboidal morphology. Static and cellular histomorphometric parameters were assessed according to the guidelines of the American Society for Bone and Mineral Research [19] using ImageJ software.

### 2.8. Fluorescent Immunohistochemical Staining

To localize CTR within the osteotomy gap, frozen sections were washed with PBS and treated with HistoReveal solution (ab103720, Abcam, Cambridge, UK). After permeabilization, sections were blocked with 3%PSA/5% donkey serum and incubated with primary anti-mouse CTR antibody (1:300, BS-0124R, Bioss, Worburn, MA, USA) overnight. Prior to the incubation with the secondary antibody (1:400, anti-rabbit, 711-165-152, Dianova, Hamburg, Germany) sections were washed in PBS. Thereafter, the slides were washed in PBS, aqua dest and mounted in Fluromount-G with DAPI (Thermofisher, Waltham, MA, USA).

### 2.9. Statistical Analysis

For all experiments, *n* = 3–6 mice per group and time point were employed. Due to loss or destruction of samples during processing, the number of available samples for analyses varied and is indicated with individual data points for each experiment. Data were analyzed by two-tailed Student’s *t*-test using GraphPad Prism software. All data are reported as mean ± standard deviation. *p* < 0.05 was considered statistically significant.

## 3. Results

### 3.1. CTR Is Expressed in Regenerating Bone

**Figure 1:**
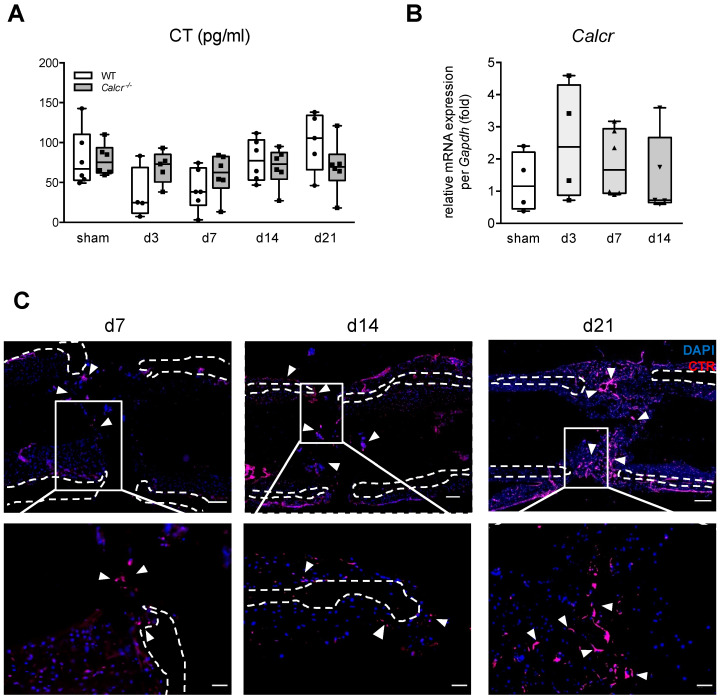
CTR is expressed in the fracture callus. (**A**) Serum CT levels in WT and CTR-deficient mice with an osteotomy and stabilized with an external fixator 3, 7, 14, and 21 days post-injury. (**B**) Gene expression of CTR (*Calcr*) in the fracture callus at the respective time points during bone regeneration. (**C**) Representative immunofluorescent images (merged) of WT callus sections 7, 14, and 21 days after surgery using a specific antibody for CTR. Dotted lines indicate cortices and arrows indicate CTR positive structures. Scale bars = 200 µm (top) and 50 µm (bottom), respectively. For (**A**,**B**) *n* = 4–6 mice per group and time point as indicated. Box plots represent median with minimum and maximum whiskers.

To study a potential role of CT/CTR signaling during bone regeneration, we first measured serum CT levels during bone healing using ELISA. Contrary to previous clinical reports [13,14], we observed a tendency towards lower circulating CT levels at the early healing stages in WT mice, which normalized later on at day 14 and day 21 following osteotomy (Figure 1A).

This trend was not present in mice lacking *Calcr* (encoding the calcitonin receptor), in which exon 6 and 7 of the *Calcr* gene are excised, rendering the receptor protein dysfunctional [7]. Monitoring gene expression in the regenerating callus, we detected robust expression of *Calcr* at all stages, with a tendency towards increased expression during early regeneration (Figure 1B). To confirm these findings on protein level, immunofluorescence on cryo-preserved callus sections from WT animals 7-, 14-, and 21-days post-injury were performed using a CTR-specific antibody. Whereas we only observed a weak signal within the osteotomy gap in WT mice 7 and 14 days after osteotomy, strong CTR expression was detected in the late remodeling stage of fracture healing at day 21 (Figure 1C). Together, although circulating CT levels were not elevated during bone regeneration, CTR was expressed at robust levels during bone healing on both mRNA and protein level. 

### 3.2. Assessment of Bone Regeneration in CTR-Deficient Mice

**Figure 2:**
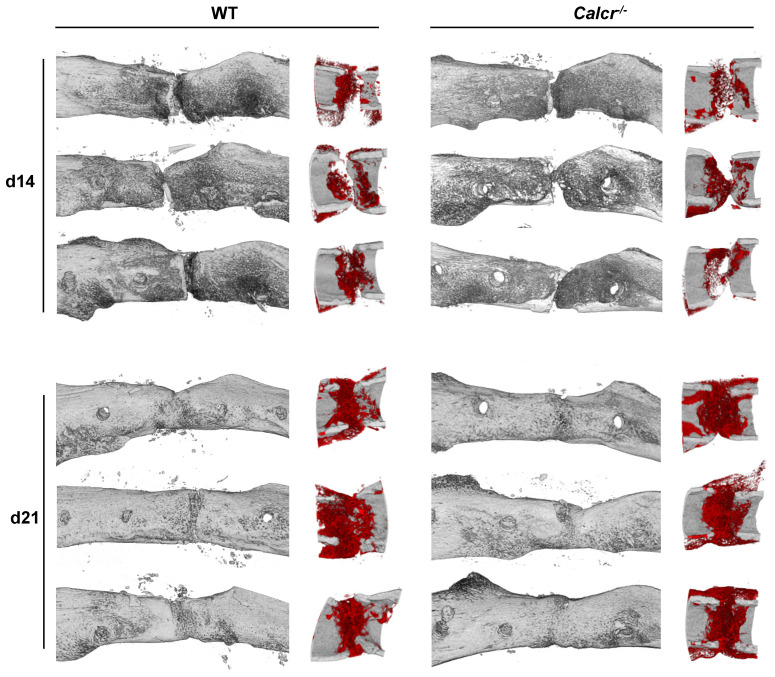
Deficiency in CTR (*Calcr^−/−^*) does not significantly affect bone regeneration. Representative μCT images (left column = sagittal overview; right column = sagittal detailed view) of the callus region in WT and *Calcr^−/−^* mice at day 14 and day 21 following osteotomy. Three representative images are shown for each group and time point. In the sagittal view, newly mineralized callus is indicated in red, whereas the original bone is displayed in grey.

In order to study a functional role of CT/CTR signaling in bone repair, we next assessed bone regeneration in mice lacking CTR (*Calcr^−/−^*) globally [7]. As we previously focused on vertebrae regarding the bone phenotype of this model, we first assessed the bone status in the femur of CTR-deficient mice using µCT in order to investigate whether the high-bone-mass phenotype is also detectable in long bones. Similar to the increased bone density in the vertebrae, CTR-deficient mice displayed increased bone volume and trabecular numbers, accompanied by a reduced trabecular separation, in the distal shaft area of the femur (Supplementary Figure S1). Thereafter, CTR-deficient and WT control mice were subjected to a femoral osteotomy, stabilized with an external fixator, and euthanized 7, 14, and 21 days post-surgery for ex vivo radiologic and histologic assessment of the healing process, covering the acute inflammation-, the soft callus- and the remodeling-stage of bone regeneration. Despite the increased bone formation in the intact skeleton in this model, radiologic analysis of the bone healing using µCT showed no improvement or alteration in regenerative capacity in CTR-deficient mice compared to controls (Figure 2).

**Figure 3:**
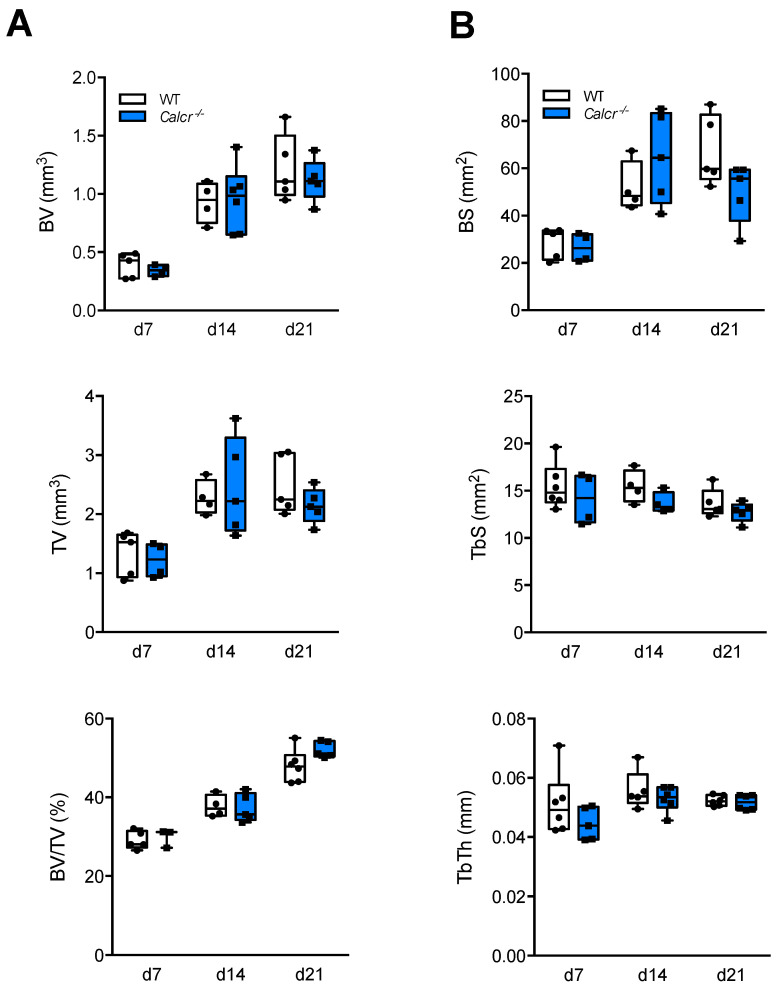
Quantification of μCT callus images. (**A**) Quantitative analysis of μCT images in mice of both genotypes at the same time points. BV = total callus bone volume (i.e., newly mineralized bone volume), TV = total tissue volume (i.e., total callus volume), BV/TV = bone volume vs. tissue volume. (**B**) Quantitative analysis of μCT images in the same samples. BS = bone surface, TbS = trabecular surface, TbTh = trabecular thickness. For (**A**,**B**) *n* = 3–6 mice per group and time point as indicated. Box plots represent median with minimum and maximum whiskers.

Quantification of respective µCT images showed that the newly mineralized bone volume, total callus volume and their ratio within the callus area did not significantly differ in mutant animals following osteotomy (Figure 3A).

Furthermore, no significant alterations in regard to callus bone surface, tissue surface, or trabecular thickness were detected in the callus of mice lacking CTR (Figure 3B).

**Figure 4:**
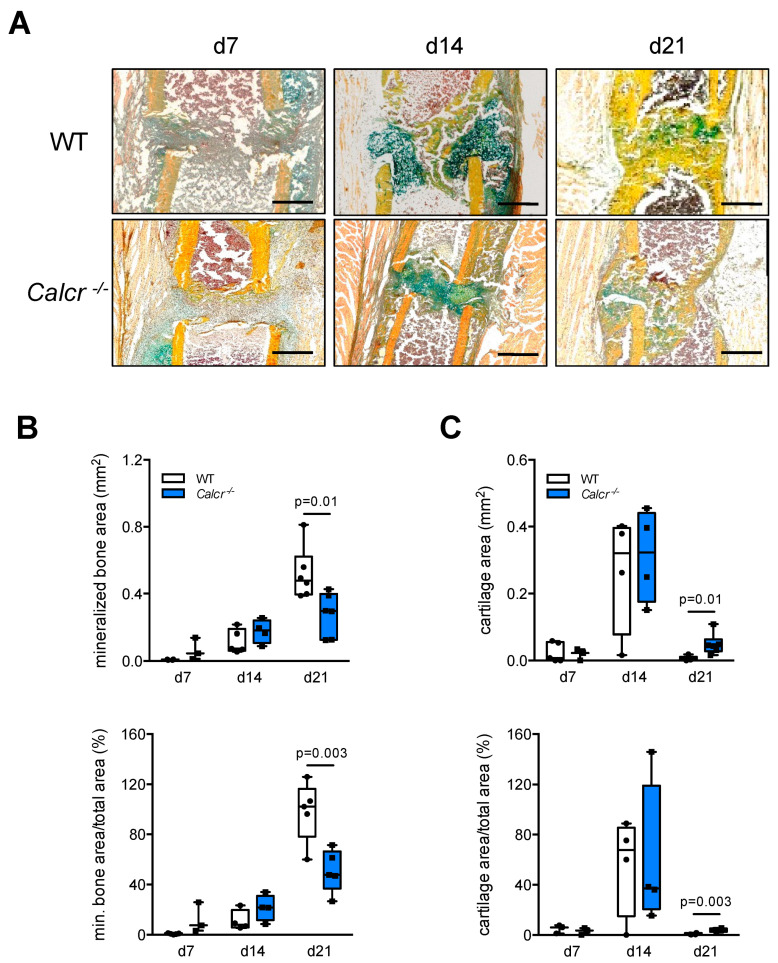
Slight alteration in callus remodeling in CTR-deficient mice. (**A**) Representative callus sections (Movat Pentachrome staining) of WT and CTR-deficient mice at the indicated time points following osteotomy (yellow = mineralized bone; green = cartilage; red = muscle). Scale bars = 500 µm. (**B**,**C**) Histomorphometric quantification of static callus parameters in the same mice. *n* = 3–6 mice per group and time point as indicated. Box plots represent median with minimum and maximum whiskers.

To investigate a possible influence of CT/CTR signaling on bone regeneration in more depth, we next performed histomorphometric characterization of the regenerating bone. For that purpose, we utilized non-decalcified, Movat Pentachrome-stained callus sections of WT and CTR-deficient mice 7, 14, and 21 days after osteotomy (Figure 4A).

Deviating from the 3D-based µCT analysis, the 2D-based, yet more sensitive histomorphometric quantification revealed a reduced amount of mineralized bone area and a lower percentage of mineralized bone area in the total callus area in mutant mice at 21 days post-surgery compared to controls (Figure 4B). Moreover, while no differences were observed at day 7 and 14, the amount of cartilage matrix in the osteotomy gap was slightly increased in CTR-deficient mice at day 21, evidenced by an alteration in cartilage area and cartilage area per total callus area when plotted against control mice (Figure 4C).

**Figure 5:**
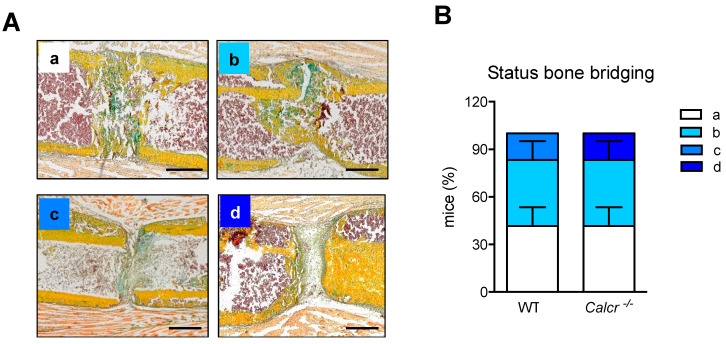
Interruption of CT/CTR-signaling does not significantly alter callus bridging. (**A**) Exemplary callus images (Movat Pentachrome staining) at day 21 following osteotomy, indicating the four different scores used for the following semiquantitative measurement of callus bridging. a = complete bridging (all four cortices bridged by callus; image derived from *Calcr^−/−^*), b = partial bridging (two to three cortices bridged by callus; image derived from *Calcr^−/−^*), c = incomplete bridging (callus present, but no bridging visible; image derived from WT), and d = nonunion (rounded cortices, minimal presence of callus; image derived from *Calcr^−/−^*). Scale bars = 500 µm. (**B**) Semi-quantitative evaluation of callus bridging in WT and CTR-deficient mice at day 21 following osteotomy in the callus sections of WT and *Calcr^−/−^* mice the indicated time points by two blinded investigators. *n* = 4–6 mice per group. Box plots represent median with minimum and maximum whiskers.

To validate the rate of fracture union and non-union in the respective mice, we performed semi-quantitative scoring of osseous callus bridging in the same callus sections exclusively at day 21 following osteotomy (Figure 5A).

In WT controls, most animals displayed complete or partial bridging of the fracture ends, while only a small percentage displayed delayed union. Bone regeneration in CTR-deficient mice was characterized by complete or partial bridging similar to what was observed in WT mice, however one individual mutant mouse displayed pronounced non-union (Figure 5B).

Collectively, the analyses of the osseous and cartilaginous tissues involved in the healing process described above showed only mild alterations in mutant mice at day 21 post-surgery, characterized by a reduction in mineralized tissue and a slightly increased amount of cartilage tissue, not affecting overall healing outcome, however.

### 3.3. Assessment of Cellular Callus Remodeling in CTR-Deficient Mice

**Figure 6:**
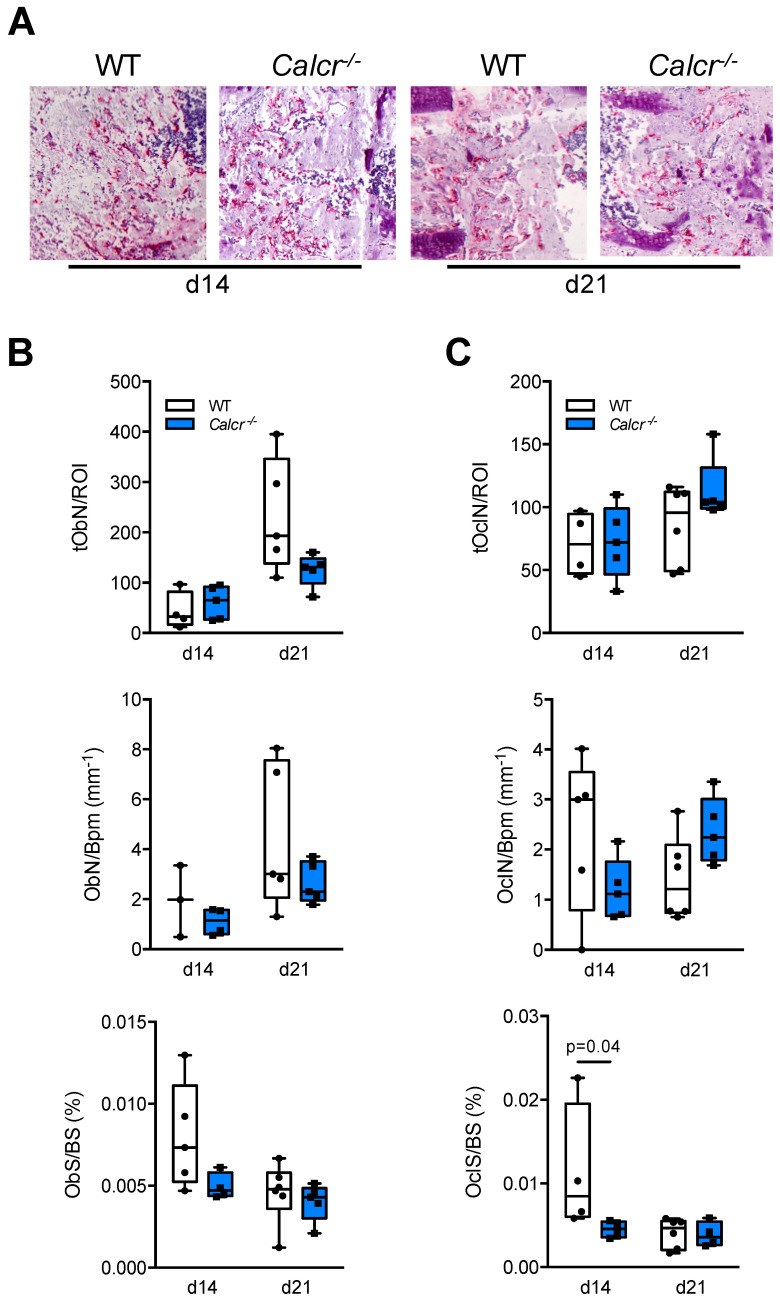
No major alteration in bone cell distribution in the callus of CTR-deficient mice (**A**) Representative TRAP-stained callus sections at day 14 and 21 in WT and CTR-deficient mice. (**B**) Histomorphometric quantification of osteoblast parameters in the callus of WT and CTR-deficient mice at the indicated time points. tObN/ROI = osteoblast numbers in the callus area, ObN/Bpm = osteoblast numbers per bone perimeter, ObS/BS = osteoblast surface per bone surface. (**C**) Histomorphometric quantification of osteoclast parameters in the callus of the same mice. tOcN/ROI = total osteoclast numbers in the callus area, OcN/Bpm = osteoclast numbers per bone perimeter, OcS/BS = osteoclast surface per bone surface. *n* = 3–6 mice per group and time point as indicated.

Following the assessment of tissue architecture during bone regeneration in mutant mice, we next investigated the cellular components of the healing process. As fully differentiated osteoblasts and osteoclasts are difficult to identify at day 7 following osteotomy, we therefore focused on cellular histomorphometry of TRAP-stained callus sections at day 14 and 21 in WT and CTR-deficient mice (Figure 6A).

Assessing osteoblast parameters, we detected neither significant differences in osteoblast numbers per callus area or per bone area, nor an alteration in osteoblast surface per bone surface (Figure 6B). Similarly, apart from a significant decrease in osteoclast surface per bone surface at day 14 following osteotomy, no significant alterations in the numbers of osteoclasts per callus area or bone area were detected at any time point (Figure 6C).

## 4. Discussion

In this study, we investigated bone regeneration in CTR-deficient mice, serving as a model for activated osteoclast-to-osteoblast coupling with an overall increased bone formation. Although we found robust CTR expression during the different stages of fracture healing, the net outcome of bone regeneration was not altered in mice lacking the CTR globally, despite some minor alteration in tissue architecture within the callus. Therefore, despite the relevance of osteoclast-to-osteoblast crosstalks in the intact skeleton, our results indicate that these processes, at least the one involving the CT/CTR/S1P axis, are only of limited relevance during the bone healing process.

Despite significant improvement in surgical techniques in the past decades, impaired bone healing, including delayed unions and non-unions, still represents an ongoing clinical challenge in orthopedic trauma surgery [20]. As treatment options remain limited [20], the understanding of the processes governing bone healing is of utmost importance in the search for therapies accelerating bone repair [21]. So far, the sole pharmaceutical molecules that gained FDA approval for clinical use promoting fracture repair are bone morphogenetic proteins (BMP) 2 and 7 [22]. However, the function of BMPs to promote bone healing is limited due to rare, but potentially severe adverse effects including heterotrophic ossification, carcinogenesis, renal and hepatic failure, and even compartment syndrome [20,21,23]. Hence, the search for suitable candidates boosting bone regeneration still remains elusive.

In this regard, the aim of this study was to investigate the impact of CT/CTR signaling on bone regeneration. Countless studies showed that pharmacologic application of CT inhibits osteoclast function and lowers systemic calcium levels [7,24]. However, we and others [7,25,26] were able to show that the physiologic function of mammalian CT is the inhibition of bone formation, as CTR-deficient mice display high bone mass and enhanced osteoblast function. Mechanistically, CTR-deficient mice display enhanced expression of the transporter protein SPNS2 in osteoclasts, resulting in enhanced release of osteoanabolic S1P into the extracellular space and increased osteoblast function [7]. In the present study, we employed CTR-deficient mice and observed only a minor impact on bone regeneration, despite their pronounced bone formation phenotype in the intact skeleton. Contrary to previous clinical reports, we did not observe an increase in circulating CT levels in WT mice in the applied osteotomy model with external fixator [13,14]. While CTR mRNA was expressed at steady levels, we observed moderate CTR protein expression during early and intermediate regeneration stages with strong signal intensities at day 21 post-surgery, pointing towards a possible role of CT/CTR signaling during bone regeneration. However, radiologic assessment of bone healing using *µ*CT imaging clearly did not demonstrate a significantly altered healing outcome in CTR-deficient mice. Histologically, only a slight influence of CT/CTR signaling could be observed, as CTR-deficient mice were characterized by a significant decrease in mineralized tissue and a significant increase of cartilage tissue at 21 days post-injury, indicating a mildly impaired healing process [27].

In light of the high bone mass phenotype of CTR-deficient mice, we are unaware of any study clearly showing that bone volume per se is a significant determiner of the rate of fracture healing. However, what clearly has been shown is that the enhancement of osteoblast activity, for example through intermittent injections of parathyroid hormone, results in improved fracture healing [28]. Likewise, the increased bone formation in leptin-deficient mice was also previously reported to result in accelerated fracture repair [29]. Thus in the current study, we hypothesized that we would also observe improved bone regeneration in mice lacking CTR as they show an enhanced bone formation rate. In our previous report on the role of the CTR in bone remodeling [7], we primarily focused our analyses on the trabecular bone architecture in the spine. In the present study, we found that the increased bone formation in CTR-deficient mice also results in increased bone mass in long bones, as we assessed fracture healing exclusively in the femur, but not in the spine. Despite their increased femoral bone volume however, CTR-deficient mice did not show improved bone regeneration. Thus, while enhanced osteoblast function may positively affect bone healing under certain circumstances, it fails to do the same in the case of increased osteoclast-to-osteoblast coupling specifically involving the CT/CTR axis.

These findings are interesting in several regards, as strong evidence is now available demonstrating the crucial role of osteoclast-to-osteoblast crosstalk in the intact skeleton. Osteoclasts release products either to enhance recruitment and differentiation of osteoblast precursors, or to stimulate osteoblast function [7,30]. Identified “osteoclast-derived coupling factors” include cardiotrophin-1 [31], Wnt10b [32], BMP6 [32], CTHRC1 [33], complement factor 3a (C3a) [34], and sphingosine-1-phosphate [7], some of which have been validated in studies using genetically modified mice. Apart from experimental approaches, the physiologic importance of this crosstalk has been clinically emphasized by the fact that anti-resorptive drugs such as bisphosphonates and denosumab not only result in an inhibition of bone resorption, but also simultaneously lead to a reduction in bone formation parameters. As bone regeneration depends on the balanced activity of osteoclasts and osteoblasts, it is thus surprising that the coupling of bone formation to bone resorption appears to be only of minor relevance during bone healing.

Our study has several limitations. First, CT/CTR signaling may exert more pronounced effects in different fracture models. As our osteotomy model is characterized by a comparatively large fracture gap, it appears possible that the crosstalk of osteoclasts and osteoblasts is impaired through the formation of a clotting fracture hematoma, rendering delicately adjusted paracrine signaling between bone cells ineffective. Second, based on the 3R principles we studied bone healing only in female mice. Thus, we are unable to conclude a possible function of CTR in male mice during bone regeneration. Finally, as the employed CTR-deficiency model is characterized by increased bone formation mediated through osteoclastic S1P, we can only speculate about the role of other identified coupling factors during bone regeneration at this stage. However, as osteoclast inhibitors including bisphosphonates and denosumab, do not affect bone healing profoundly [35], it appears that efferent signaling from osteoclasts to osteoblasts is indeed only of secondary importance during bone regeneration.

## 5. Conclusions

In conclusion, despite the essential role of CT/CTR signaling in regulating bone formation in the intact skeleton through S1P, our data indicate that this pathway does not significantly affect bone regeneration, at least in female mice with the employed osteotomy model. Future studies are warranted to mechanistically dissect the differential impact of osteoclast-to-osteoblast coupling during varying musculoskeletal conditions.

## Data Availability

All relevant data is presented in the manuscript; raw data is available upon request from the corresponding author.

## Supplementary Materials

The following are available online at https://www.mdpi.com/article/10.3390/cells10092304/s1, Figure S1: High bone mass in the distal femur of mice lacking CTR.
