# Supplementary material for: Mice Lacking the Calcitonin Receptor Do Not Display Improved Bone Healing
Source: Cells. 2021 Sep 3;10(9):2304. doi: 10.3390/cells10092304 (PMC8471896; doi:10.3390/cells10092304)
Supplement: Supplementary file 1 [file cells-10-02304-s001.zip › cells-1309268-supplementary.pdf]

Supplementary materials:

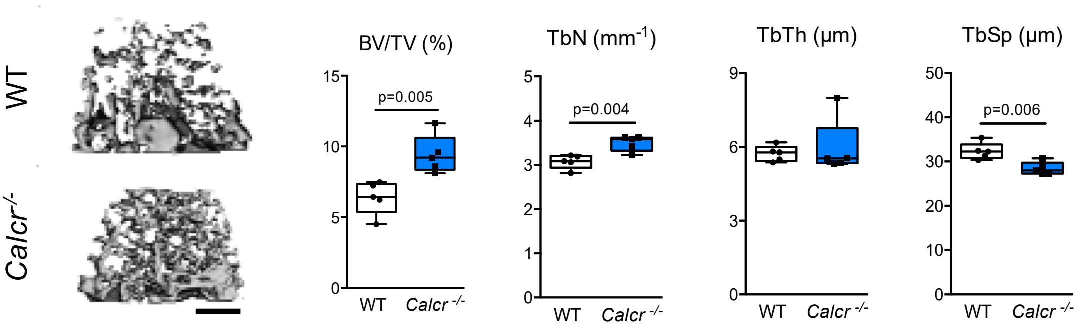

Supplementary Figure S1. High bone mass in the distal femur of mice lacking CTR. Representative µCT images of the trabecular bone in the distal shaft area of the femur derived from untreated WT and CTR-deficient (*Calcr*$^{-/-}$) mice at the age of 14 weeks (left; scale bar = 500 µm.), and radiologic quantification of indicated bone parameters (right). n = 5 mice per group.
